# Transcriptomic Abnormalities in Epstein Barr Virus Associated T/NK Lymphoproliferative Disorders

**DOI:** 10.3389/fped.2018.00405

**Published:** 2019-01-17

**Authors:** Sanjay de Mel, Joshua Zhi-Chien Tan, Anand D. Jeyasekharan, Wee-Joo Chng, Siok-Bian Ng

**Affiliations:** ^1^Department of Haematology-Oncology, National University Cancer Institute of Singapore, National University Health System, Singapore, Singapore; ^2^Department of Medicine, National University Health System, Singapore, Singapore; ^3^Cancer Science Institute of Singapore, National University of Singapore, Singapore, Singapore; ^4^Department of Pathology, Yong Loo Lin School of Medicine, National University of Singapore, Singapore, Singapore; ^5^Department of Pathology, National University Health System, Singapore, Singapore

**Keywords:** Epstein–Barr virus, extranodal NK/T cell lymphoma, chronic active EBV infection, T cell lymphoma, RNA

## Abstract

Epstein Barr virus positive T/NK lymphoproliferative disorders (EBV-TNKLPD) comprise a spectrum of neoplasms ranging from cutaneous lymphoid proliferations to aggressive lymphomas. The spectrum includes extranodal NK/T-cell lymphoma (ENKTL), aggressive NK-cell leukemia, and a group of EBV-TNKLPDs affecting children which are poorly characterized in terms of their molecular biology. Gene and miRNA expression profiling has elucidated RNA abnormalities which impact on disease biology, classification, and treatment of EBV-TNKLPD. Pathways promoting proliferation, such as Janus associated kinase/ Signal Transducer and Activator of Transcription (JAK/STAT) and nuclear factor kB, are upregulated in ENKTL while upregulation of survivin and deregulation of p53 inhibit apoptosis in both ENKTL and chronic active EBV infection (CAEBV). Importantly, immune evasion via the programmed cell death-1 and its ligand, PD-1/PD-L1 checkpoint pathway, has been demonstrated to play an important role in ENKTL. Other pathogenic mechanisms involve EBV genes, microRNA deregulation, and a variety of other oncogenic signaling pathways. The identification of EBV-positive Peripheral T-cell lymphoma not otherwise specified (PTCL-NOS) as a tumor with a distinct molecular signature and clinical characteristics highlights the important contribution of the knowledge derived from gene and miRNA expression profiling in disease classification. Novel therapeutic targets identified through the study of RNA abnormalities provide hope for patients with EBV-TNKLPD, which often has a poor prognosis. Immune checkpoint inhibition and JAK inhibition in particular have shown promise and are being evaluated in clinical trials. In this review, we provide an overview of the key transcriptomic aberrancies in EBV-TNKLPD and discuss their translational potential.

## Introduction

Epstein–Barr virus (EBV) is a ubiquitous human herpesvirus with B-cell tropism and the ability to transform infected B lymphocytes into continuously proliferating lymphoblastoid cells. Infrequently, EBV infects T cells and Natural Killer (NK) cells, which can result in a wide spectrum of EBV-positive cytotoxic T/NK cell lymphoproliferative diseases (EBV-TNKLPD). The current classification of EBV-TNKLPD includes (i) systemic chronic active EBV infection of T- and NK-cell type (CAEBV), (ii) cutaneous CAEBV, which includes hydroa vacciniforme-like lymphoproliferative disorder (HV-LPD) and severe mosquito bite allergy (MBA), (iii) systemic EBV-positive T-cell lymphoma of childhood (STCL), (iv) aggressive NK-cell leukemia (ANKL), (v) extranodal NK/T-cell lymphoma, nasal-type (ENKTL), and (vi) nodal peripheral T-cell lymphoma, EBV-positive (EBV-PTCL) (1–3). ENKTL and ANKL are the prototypic examples of EBV-TNKLPD and are well-recognized (4). On the other hand, the classification of EBV-TNKLPD occurring in childhood (EBV-TNKLPD-childhood) has evolved and was recently updated in the revised 4th edition of the WHO classification (1). It includes a spectrum of diseases with heterogeneous clinical manifestations, a broad range of morphology from polymorphic to monomorphic lymphoid proliferations, and indolent behavior to systemic and aggressive diseases (5). EBV-TNKLPD involving primarily lymph nodes is uncommon but shows characteristic clinical and molecular features distinct from ENKTL (6). This group of nodal EBV-TNKLPD is currently classified as an EBV-positive variant of PTCL, not otherwise specified (EBV-PTCL), as it is presently unclear whether they represent a distinct entity (2).

The spectrum of EBV-TNKLPD remains a challenging group of diseases to study and this is often attributed to the rarity of disease and limited tissue availability (7, 8). Due to significant overlap in morphology and phenotype, the precise distinction of each of the entities can be challenging (9). Furthermore, the nomenclature and classification of EBV-TNKLPD occurring in childhood has been confusing and has suffered from a lack of well-established diagnostic criteria until recently (3, 10).

**Figure 1:**
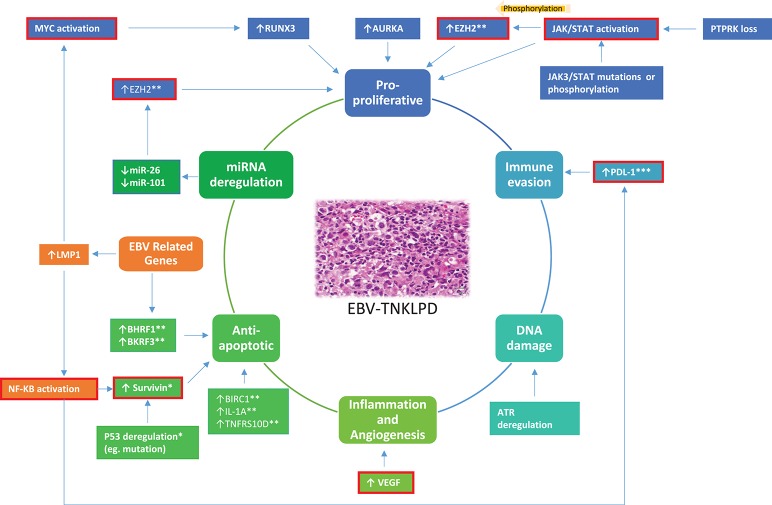
A proposed model highlighting an overview of the main RNA abnormalities in EBV-TNKLPD. The key processes involved in the lymphomagenesis of EBV-TNKLPD are indicated in the central circle of the figure. The RNA abnormalities are grouped and color-coded according to the postulated processes and those which are likely to contribute to multiple processes are highlighted in orange. RNA abnormalities which have potential translational impact are outlined with red. All the above RNA abnormalities are present in ENKTL. ^*^Refers to RNA abnormalities which are present in ENKTL, Systemic EBV+ T-Cell Lymphoma and CAEBV. ^**^Refers to RNA abnormalities which are present in ENKTL and CAEBV. ^***^Refers to RNA abnormalities which are present in ENKTL and EBV+ PTCL NOS.

Genome-wide gene expression profiling (GEP) has revolutionized and greatly improved our understanding of the molecular biology of lymphoma (11, 12). Similarly, GEP has identified robust molecular signatures for subtypes of PTCL and ENKTL and uncovered actionable therapeutic targets (13, 14). Novel insights gleaned from recent genome-wide high throughout techniques have also significantly advanced our understanding of ENKTL (8, 15–18). On the other hand, knowledge of the molecular biology and genomics of EBV-TNKLPD of childhood (EBV-TNKLPD-childhood), such as CAEBV, is only slowly unraveling and there remains a paucity of large-scale gene or miRNA profiling of the diseases in childhood. In this brief review, we will summarize current insights on the transcriptomics abnormalities in EBV-TNKLPD and focus on those with translational impact on (i) understanding molecular biology and disease pathogenesis, (ii) disease classification and refining diagnosis, (iii) disease prognosis, and (iv) therapy. An overview of these abnormalities and their role in the pathogenesis of EBV-TNKLPD is presented in Figure 1.

## Impact of Transcriptomic Abnormalities on Understanding Disease Pathogenesis or Deregulated Pathways

### Cellular Proliferation

Janus Kinase/Signal Transducer and Activator of Transcription (JAK/STAT) signaling has recently been shown to play a prominent role in the pathogenesis of ENKTL and ANKL (19, 20). GEP studies have revealed that components of this pathway are differentially expressed in ENKTL compared to normal NK cells (15, 21). Transcriptomic sequencing and integrated genomic analysis of ANKL showed that JAK/STAT mutations resulted in overexpression of MYC and its interacting proteins (20). These data suggest that JAK/STAT signaling promotes proliferation of malignant NK cells not only through its known pro-proliferative function but also through interaction with oncogenes, such as MYC. MYC is upregulated in ENKTL, and this is associated with a corresponding overexpression of its transcriptional targets. (17). Among these MYC targets is RUNX3, which promotes proliferation and survival in ENTKL (22). Inhibition of MYC leads to downregulation of RUNX3 and apoptosis in ENTKL cells, which supports a potential therapeutic role of MYC in ENKTL (22).

Enhancer of Zeste Homolog 2 (EZH2), a component of the polycomb repressive complex 2 (PRC2), has also been shown to be upregulated in ENKTL (17, 23). This may be explained by Myc-induced downregulation of microRNAs, miR-26a, and miR-101, which negatively regulate EZH2 expression (23). In ENKTL, EZH2 does not function as an epigenetic regulator. Instead, it acts as a transcriptional co-activator via a non-canonical pathway (24), and this switch of function is mediated by JAK3 via phosphorylation of EZH2. Inhibition of JAK3 has been demonstrated to reduce the proliferation of ENKTL cells, indicating that targeting this pathway may be a potential therapeutic strategy (25). EZH2 has also been shown to be upregulated in EBV-TNKLPD-childhood and, similar to ENKTL, downregulation of EZH2 using a PRC2 inhibitor induces apoptosis in CAEBV cell lines (23, 26).

Nuclear factor (NF) kB is a transcription factor with pro-survival and anti-apoptotic functions known to be upregulated in lymphoid malignancies (27). NF-kB and its target genes were shown to be overexpressed in ENKTL by GEP in two studies (15, 17) and treatment of ENKTL cell lines with NF-kB inhibitors resulted in induction of apoptosis (28). However, these results were not replicated in another study comparing ENKTL to PTCL (16). Aurora kinase A (AURKA) is a mitotic kinase important for cell proliferation. AURKA is also overexpressed in ENKTL and targeted inhibition induced significant growth arrest in ENKTL cell lines (16, 17). Recent data suggest that AURKA interacts with MYC and WNT pathways to promote cell proliferation (29, 30). Further studies are required to better delineate the role of AURKA in ENKTL pathogenesis.

Murakami et al. performed GEP on CAEBV and ENKTL samples and identified upregulation of interleukin-2 (IL-2), IL-10, interferon gamma receptor 1 (IFNGR1), and Inhibin beta A (INHBA) (31). It has been proposed that EBV-infected T cells secrete IL-2 which functions via an autocrine loop to promote proliferation of T-cells in CAEBV. The binding of IFN-γ to IFNGR1 leads to activation of the JAK-STAT pathway, which is amenable to targeted therapy. (32, 33) The precise role of INHBA and IL-10 in EBV-TNKLPD remains unknown (31). Other genes that may confer a proliferative signature and are upregulated in ENKTL and CAEBV cell lines include cyclin dependent kinase 2 (CDK2), a regulator of cell cycle progression, and heat shock 90kDa protein 1-alpha (HSPCA), which is important for normal protein folding and survival of cancer cells (34).

In summary, there are multiple pathways which may provide mitogenic signals and allow the neoplastic cells to survive and proliferate in ENKTL and CAEBV. Among the pathways described above, JAK/STAT and NF-kB are the best studied in ENKTL. EZH2 and AURKA have promising translational impact and may serve as potential therapeutic targets in ENKTL. The evaluation of JAK3 inhibitors as modulators of non-canonical EZH2 activity in clinical trials is warranted.

### Deregulation of Apoptosis and the DNA Damage Response

Resistance to apoptosis is a known hallmark of cancer and development of drug resistance (35). Several protein families that act as negative regulators of apoptosis by inhibiting cell death signaling pathways have been reported to be upregulated in CAEBV and ENKTL, including BIRC1, interleukin 1 alpha (IL1A), tumor necrosis factor receptor superfamily member 10d (TNFRS10D), survivin, p53, and NF-kB (15, 17, 26, 34, 36). Survivin is an anti-apoptotic protein that is overexpressed in the majority of ENKTL and EBV-TNKLPD-childhood (17, 26). Treatment of ENKTL cells with a survivin inhibitor, terameprocol, leads to increased apoptosis, suggesting this may be a therapeutic target. p53 is upregulated in ENKTL and EBV-TNKLPD-childhood and this is associated with deregulation of genes which are normally controlled by p53 (17, 26). p53 deregulation in ENKTL may be caused by mutations as reported by Quintanilla-Martinez et al. (36).

Appropriate response to DNA damage is essential for the maintenance of genome stability. Ataxia-telangiectasia mutated (ATM) and ataxia-telangiectasia-related (ATR) kinases are central regulators of the DNA damage response signaling pathway (37). Deregulation of ATR protein has been reported in ENKTL and CAEBV due to deletions resulting from aberrant splicing and leads to an abnormal response to DNA damage (38). ATR and related cell cycle checkpoint genes were also found to be overexpressed in ENKTL in another study by Ng et al. (17).

These data suggest that a defective DNA damage response along with inhibition of apoptosis may contribute to lymphomagenesis of EBV-TNKLPD. Survivin and p53 stand out as key players in the pathogenesis of EBV-TNKLPD. Survivin in particular may have potential as a therapeutic target based on the pre-clinical data discussed. Non-apoptotic cell death pathways are also a potential research arena for drug discovery and targeted therapies and are especially important for the circumvention of drug resistance (35).

### Immune Evasion

Programmed cell death-1 (PD-1) inhibition has changed the landscape of immunotherapy for lymphoid malignancies (39, 40). GEP studies have shown that PD-Ligand 1 (PD-L1, also known as CD274) mRNA is upregulated in ENTKL compared to control tissues (6, 8). Overexpression of PD-L1 in ENKTL has been proposed to be mediated by LMP1 via MAPK, NF-kB, and STAT3 signaling (41, 42). The glycoprotein, CD38, is expressed in the majority of ENKTL and its expression is associated with an inferior outcome (8, 43). We have demonstrated through GEP study that CD38 is upregulated in ENKTL compared to control tissues (GEO database GSE90597) (8). Recent *in vitro* studies revealed that daratumumab, a humanized monoclonal antibody approved for the treatment of relapsed multiple myeloma, has good efficacy against ENKTL (44). Our current understanding of the role of PD1 and CD38 in EBV-TNKLPD remains incomplete. Novel regulators of PD1 such as CMTM6 (45) warrant investigation in this context while the function of CD38 in lymphomagenesis requires further study.

Whole-transcriptome microarray studies have identified a unique set of 30 genes which are dysregulated in CAEBV (46). These include several phagocytosis-associated genes such as C1QC, FGL232, and PSTPIP233 as well as monocyte markers FCGR1A and FCGR1B (CD64A/B), suggesting a relatively hyperactive phagocytosis and monocyte-mediated antibody-dependent cellular cytotoxicity in CAEBV (46). The expression of many CAEBV-unique genes was highly correlated with the level of CD64, indicating an important role for monocytes in the cellular immune response to CAEBV (46). Understanding the immune microenvironment of EBV-TNKLPD will be helpful in the incorporation of immunotherapy in this group of diseases. The PD-1/PD-L1 pathway is the most important transcriptomic abnormality from a biological and translational point of view. The potential of this pathway as a therapeutic target is discussed below.

### Tumor Promoting Inflammation and Angiogenesis

Chronic inflammation is a known driver of malignancy and angiogenesis is critical for tumor growth and metastasis (47). Vascular endothelial growth factor (VEGF) promotes tumor vascularization and growth in a variety of malignancies (48). VEGF is upregulated in ENKTL and has been proposed as a therapeutic target (7, 49). Guanylate-binding protein 1 (GBP1), a G protein involved in the chronic inflammatory response and strongly induced in endothelial cells and lymphocytes, was found to be overexpressed in CAEBV cells (50). It is postulated that the upregulation of IFNGR1 in CAEBV may result in the overexpression of GBP1, which in turn contributes to vascular dysfunction in chronic inflammation (31). Tumor necrosis factor alpha-induced protein 6 (TNFAIP6) is an adhesion molecule that plays multiple roles in chronic inflammation and tissue remodeling. TNFAIP6 is upregulated in CAEBV and postulated to play a similar role to GBP1 in this context (50). Activated T-cells in CAEBV express higher levels of interleukin-10 (IL-10), transforming growth factor-β (TGF-β), and IFN-γ (51), with the expression of IL-10 and TGF-β being proportional to the EBV viral load in T cells (51). These data suggest that a complex deregulation of pro-inflammatory cytokines driven by EBV as well as a potent angiogenic drive play a crucial role in the pathogenesis of EBV-TNKLPD. VEGF appears to have the greatest translational potential among the deregulated angiogenic pathways discussed and requires further study.

### EBV Related Genes

EBV mediated oncogenesis is thought to be driven by genes expressed during latency, such as LMP1 (52). The expression of EBV-related lytic genes, such as BHRF1 and BKRF3, was found to be increased in ENKTL cell lines and may have an anti-apoptotic role as BHRF1 has sequence homology with human BCL-2 (34). BZLF1, which encodes the immediate-early gene product Zta, was preferentially expressed in CAEBV compared to ENKTL cell lines (34). Given the critical role of EBV, further studies are required to fully understand the mechanistic underpinnings of the virus in the lymphomagenesis of this spectrum of disease with the aim of developing therapeutic targets.

### Other Oncogenic Signaling Pathways

Other signaling pathways reported to play a pathogenic role in ENKTL include PDGFRα, AKT, and NOTCH-1 (7, 15). The availability of inhibitors to the NOTCH and AKT signaling pathways is currently under evaluation, and their clinical efficacy in ENKTL remains to be established (53, 54).

### MicroRNA Deregulation

MicroRNAs (miRNA) have a critical role in the regulation of gene expression in cancer and have been proposed to play an important role in ENKTL and CAEBV (55, 56). miRNAs in ENKTL are predominantly downregulated compared to normal NK cells, specific examples include miR-150, miR-101, miR-26a, miR-26b, miR-28-5, miR-363, and miR-146 (57, 58). The targets of these miRNAs include genes in critical pathways such as p53, MAPK, and EZH2 (23, 57). Less commonly, miRNAs with pro-oncogenic functions, such as miR-21 and miR-155, are upregulated in ENKTL (59). miRNA profiles have also been suggested to have prognostic significance. For instance, downregulation of miR-146a and upregulation of miR221 are associated with poor prognosis (60, 61). The utility of miRNAs as a therapeutic target is, however, unknown and requires further studies.

Less is known about the miRNA profile of CAEBV than ENKTL. In a study of patients with CAEBV compared to those with infectious mononucleosis and healthy controls, miR-BART 1-5p, 2-5p, 5, and 22 were found to be upregulated in CAEBV patients (62). Interestingly, miR-BART 13, miR-BART 2-5p, and 15 levels were higher in patients with active compared to inactive disease, suggestive of a potential role in monitoring disease activity (56, 62).

## Impact of Transcriptomic Abnormalities on Disease Classification and Refining Diagnosis

In addition to characterizing deregulated oncogenic pathways, GEP studies have provided new perspectives on the molecular biology, ontogeny, and classification of ENKTL. The GEP of ENKTL is distinct from PTCL NOS and shows higher expression of genes associated with NK cell lineage (14, 15). In addition, ENKTL demonstrated a similar molecular signature to a subset of gamma-delta (γδ) T-cell lymphomas that are non-hepatosplenic in presentation and akin to a subset of ENKTL derived from γδ T-cells (16). Interestingly, these “molecularly defined” γδ T-cell lymphomas had similar clinical outcomes to ENKTL (14, 16).

EBV-PTCL is an EBV-associated T/NK cell lymphoma with primary nodal disease presentation and shows molecular and clinical features distinct from ENKTL, including older age, lack of nasal involvement and CD8-positive/CD56-negative phenotype (6). Gene set enrichment analysis revealed significant enrichment for hallmark gene sets, such as MTORC1_SIGNALING, IL6_JAK_STAT3_SIGNALING as well as several gene sets related to cell cycle and genomic instability, including G2M_CHECKPOINT, E2F_TARGETS, MYC_TARGETS, and APOPTOSIS. Current data support the WHO proposal to classify this disease separately from ENKTL and this distinction is clinically important as EBV-PTCL is significantly more aggressive than ENKTL and should be managed differently (6).

Since there is significant overlap in the clinicopathologic features of ENKTL and EBV-TNKLPD-childhood, studies have also been conducted to compare the molecular signature of these entities. GEP results indicated a high degree of similarity between EBV-TNKLPD-childhood and ENKTL, with overexpression of p53, survivin, and EZH2 (26). Notably, there is a distinctive enrichment of stem cell-related genes in EBV-TNKLPD-childhood compared to ENKTL (26). The discovery of potential cancer stem cell phenotype in EBV-TNKLPD-childhood has potential therapeutic implications and may explain why conventional chemotherapy, without hematopoietic stem cell transplant, is often unsuccessful in the treatment of the disease in childhood (63, 64).

## Impact of Transcriptomic Abnormalities on Disease Prognostication

The challenge in EBV-TNKLPD-childhood remains to identify morphological or clinical markers to predict outcome. While age, liver dysfunction, and treatment with transplantation play a role in prognosis (65), criteria such as presence of systemic symptoms, T-cell clonality, amount of EBV-positive cells, and/or density of the infiltrate do not help in predicting disease progression (66). In this regard, Ng et al. compared the molecular signature between 2 groups of EBV-TNKLPD-childhood with different outcomes and identified overexpression of cyclinE2 gene and protein to be significantly associated with poor outcome (67).

The prognostication of ENKTL is largely based on clinical features (68). Transcriptomics abnormalities have not made a significant contribution to the risk stratification of ENKTL although there is a suggestion that specific miRNAs (miR-146a and miR-221) may have a prognostic impact (60, 61).

## Impact of Transcriptomic Abnormalities on Therapy

Transcriptomics aberrancies in EBV-TNKLPD have provided insight into potential therapeutic targets. Among the most promising is immune checkpoint inhibition. Overexpression of PD-L1 mRNA and protein has been demonstrated in ENKTL and EBV-PTCL (6, 8) and patients with relapsed ENKTL have shown remarkable response to pembrolizumab, an antibody against PD1 (69). However, there is a lack of correlation between clinical response to pembrolizumab with PD-L1 expression in tumor cells. Further research is therefore required to understand the mechanism of action and to identify new predictive biomarkers for checkpoint inhibition.

The anti-CD38 monoclonal antibody, daratumumab, is approved for multiple myeloma (70). Recent *in vitro* studies revealed that daratumumab may also have efficacy against ENKTL (44). This hypothesis was supported clinically by the dramatic response of a patient with refractory ENKTL to daratumumab monotherapy (71). Targeting LMP1/2 using cytotoxic T-lymphocytes has also revealed encouraging results in EBV-associated lymphomas. While these data have shown initial promise, further study is required to evaluate the mechanism of action and clinical efficacy of these agents (40).

The JAK-STAT pathway is another potentially useful therapeutic target with JAK3 and STAT3 inhibitors showing *in vitro* activity against ENKTL (25, 72). JAK inhibitors are currently being evaluated against ENKTL in a phase 2 clinical trial (NCT02974647) (73). The potential for JAK inhibition to target the INF-γ pathway in CAEBV is also an attractive therapeutic option requiring further study (31). Proteasome inhibitors to target NF-kB have been evaluated in clinical trials in combination with chemotherapy and additional evaluation is necessary to assess their efficacy in ENKTL (74, 75). Other potential therapeutic targets based on preliminary *in vitro* data on ENKTL include PDGFRa, VEGF, AURKA, NOTCH, CDK2, MYC, EZH2, and survivin (7, 8, 15–17, 22, 23).

## Conclusions and Future Directions

Understanding gene and miRNA transcriptomic abnormalities in EBV TNKLPD has improved our understanding of the molecular biology of this group of tumors, with an impact on disease classification, prognosis, and treatment. The key abnormalities associated with each entity are summarized in Table 1 and an overview of the major deregulated pathways is represented in Figure 1. Further characterization of the molecular signatures of these tumors, especially those occurring in childhood, will help direct functional studies on the disease pathogenesis and decipher the role of EBV while stimulating insights into development of new treatment strategies for these patients.

**Table 1:** Transcriptomic abnormalities in EBV associated NK and T lymphoproliferative disorders.

**Transcriptomic abnormality**	**Role in lymphoma biology**	**Subtypes of EBV+TNKLPD**	**References**	**Clinical significance for therapeutics**	**References**
JAK/STAT	Upregulated via mutation or phosphorylation. Transcriptomic sequencing and integrated genomic analysis of ANKL showed that JAK/STAT mutations resulted in overexpression of MYC and its interacting proteins.	ENKTL ANKL	(20); (8)	Anti-tumor activity of JAK-3 and STAT-3 inhibition in pre-clinical/*in vitro* models. Clinical trials evaluating JAK inhibitors in ENKTL ongoing.	(25); (72)
RUNX3	Upregulated and has oncogenic role promoting proliferation and survival in ENTKL.	ENKTL	(22)	MYC inhibition *in vitro* leads to down-regulation of RUNX3 and apoptosis, suggesting MYC as potential therapeutic target.	(22)
EZH2	Upregulated and functions as a transcriptional co-activator via a non-canonical pathway in ENKTL.	ENKTL and systemic EBV+T-cell lymphoma	(24); (26)	Targeting EZH2 using a PCR2 inhibitor induces apoptosis in ENKTL.	(23); (17)
NF-kB	Upregulated and promotes survival and proliferation.	ENKTL	(15); (17)	Bortezomib in ongoing early phase clinical trials for ENKTL.	(28); (74); (75)
AURKA	Upregulated, promotes cell proliferation.	ENKTL	(16); (17)	*in vitro* inhibition of AURKA induced apoptosis	(16)
IL-2	Upregulated. Promotes T-cell proliferation.	ENKTL and CAEBV	(31)	N/A	N/A
IL-10	Upregulated. Precise role unclear.	ENKTL and CAEBV	(31)	N/A	N/A
IFNGR1	Upregulated. Binding of IFN-γ activates JAK-STAT pathway.	ENKTL and CAEBV	(31)	N/A	N/A
INHBA	Upregulated. Promotes survival and inhibits apoptosis of EBV infected T-cells.	ENKTL and CAEBV	(31)	N/A	N/A
CDK2, HSPCA	Upregulated. Promotes proliferation and survival of cancer cells.	ENKTL and CAEBV	(34)	N/A	N/A
BIRC1, IL-1A, TNFRS10D	Upregulated, inhibits apoptosis.	ENKTL and CAEBV	(34)	N/A	N/A
Survivin	Upregulated. Inhibits apoptosis.	ENKTL, Systemic EBV+T-cell Lymphoma and CAEBV	(17); (26)	Survivin inhibition *in vitro* induced apoptosis, suggesting potential therapeutic role.	(17); (8)
P53	Upregulated (e.g., by mutation). Inhibits apoptosis.	ENKTL, Systemic EBV+T-cell Lymphoma and CAEBV	(17); (36)	N/A	N/A
ATR	Deregulation (e.g., deletion) resulting in abnormal DNA damage response.	ENKTL	(38)	N/A	N/A
PD-L1	Upregulated. Involved in immune evasion.	ENKTL and EBV+PTCL NOS	(6); (8)	Patients with relapsed ENKTL showed response to pembrolizumab, an antibody against PD1. No data yet on EBV+PTCL NOS	(69)
CD38	Upregulated. Exact role unknown but associated with poorer prognosis.	ENKTL	(40); (8)	Good *in vitro* efficacy of daratumumab and one case report documenting complete response.	(44); (71)
VEGF	Upregulated. Promotes tumor vascularization and growth.	ENKTL	(49); (8)	Potential therapeutic target.	(49)
EBV lytic genes (BHRF1, BKRF3, BZLF1)	Upregulated. Potential pathogenic role in ENKTL and CAEBV. BHRF1 may have anti-apoptotic role due to sequence homolog to human BCL-2.	ENKTL and CAEBV	(34)	N/A	N/A
PDGFRα	Upregulated. Mediates migration, proliferation, and cell survival.	ENKTL	(8)	Potential therapeutic target for tyrosine kinase inhibitors.	(15)
NOTCH	Upregulated in ENKTL, involved in developmental processes and cancer.	ENKTL	(15)	Potential therapeutic target for NOTCH inhibitors.	(54)
miR-150 miR-101 miR-26a miR-26b miR-28-5 miR-363 miR-146	Downregulated of miRNAs in ENKTL. Targets of these miRNAs include genes in critical pathways such as p53, MAPK and EZH2	ENKTL	(57)	N/A	N/A
miR-21 miR-155	Upregulated and have a pro-oncogenic function	ENKTL	(59)	N/A	N/A
miR-146a	Downregulated, associated with poor prognosis	ENKTL	(60)	N/A	N/A
miR-221	Upregulated, associated with poor prognosis.	ENKTL	(61)	N/A	N/A
C1QC FGL232 PSTPIP233 FCGR1A (CD64A) FCGR1B (CD64B)	Dysregulation, suggesting a relatively hyperactive phagocytosis and monocyte-mediated antibody-dependent cellular cytotoxicity (ADCC) in CAEBV.	CAEBV	(46)	N/A	N/A
GBP1	Upregulated. Contributes to vascular dysfunction in chronic inflammation.	CAEBV	(31); (50)	N/A	N/A
TNFAIP6	Upregulated, Multiple roles in chronic inflammation and tissue remodeling.	CAEBV	(50)	N/A	N/A
IL-10 TGF-β IFN-γ	Higher levels expressed in T cells in CAEBV. May be a viral evasion mechanism in CAEBV.	CAEBV	(51)	N/A	N/A
LMP1	LMP1 expressed in CAEBV and promotes proliferation.	CAEBV	(52)	N/A	N/A
miR-BART 1-5p miR-BART 2-5p miR-BART 5 miR-BART 22	Upregulated.	CAEBV	(62)	N/A	N/A
miR-BART 2-5p miR-BART 13 miR-BART 15	Upregulated in patients with active compared to inactive disease.	CAEBV	(56); (62)	N/A	N/A

*ENKTL, Extranodal NK Tcell lymphoma; EBV, Epstein Barr Virus; CAEBV, Chronic Active EBV; PTCL NOS, Peripheral T-cell lymphoma not otherwise specified; ANKL, Aggressive NK cell leukemia; N/A, No available data to support a therapeutic role at present*.

## Author Contributions

S-BN and W-JC conceptualized the review. SdM and S-BN wrote the manuscript. JT prepared the figure and table. AJ and W-JC critically reviewed and edited the manuscript, figure, and table.

### Conflict of Interest Statement

The authors declare that the research was conducted in the absence of any commercial or financial relationships that could be construed as a potential conflict of interest.
